# Secondary Erythrocytosis Associated with Uterine Myoma Is Rare but Should Be of Concern

**DOI:** 10.1155/2023/7520453

**Published:** 2023-03-17

**Authors:** Ekasak Thiangphak, Ingporn Jiamset, Phawat Matemanosak, Athithan Rattanaburi

**Affiliations:** Department of Obstetrics and Gynecology, Faculty of Medicine, Prince of Songkla University, Songkhla, Thailand

## Abstract

Myomatous erythrocytosis syndrome (MES) is a rare gynecological condition, defined by the presence of the clinical triad of erythrocytosis, uterine fibroids, and normalization of red blood cell counts after the surgical removal of uterine fibroids. Herein, we report the case of a woman, in the postmenopausal stage, with the clinical triad of MES. She had a history of erythrocytosis of unknown etiology and underwent phlebotomy for a year prior to visiting our hospital. Pre-operative hemoglobin (Hb) level, hematocrit (Hct) level, and red blood cell (RBC) count were 18.1 g/dL, 56.1%, and 6.52 million cells/*μ*L, respectively. She underwent exploratory laparotomy, transabdominal hysterectomy, and bilateral salpingo-oophorectomy. The operative findings revealed a large uterine myoma, and the pathology result was compatible with uterine leiomyoma. All hematologic parameters returned to the normal range on post-operative day 1. Her hematologic parameters returned to normal values 4 weeks after surgery with a Hb level of 13.5 g/dL, Hct level of 41.2%, and RBC count of 4.92 million cells/*μ*L. The exact pathophysiology of this condition remains unknown. However, surgical removal of uterine myoma is the mainstay of treatment. Despite the rarity of this condition, its diagnosis should be considered in patients presenting with erythrocytosis and uterine masses.

## 1. Introduction

Erythrocytosis, also known as polycythemia, is a condition that increases the number of red blood cells (RBCs) or RBC masses. In women, hemoglobin (Hb) levels >165 g/L or hematocrit (Hct) >48% are indicative of this condition [1]. Erythrocytosis can be classified as primary, in which there is increasing RBC production in the bone marrow, and secondary if there are other causes outside the bone marrow [1]. Myomatous erythrocytosis syndrome (MES) is a secondary erythrocytosis caused by uterine fibroids. To date, only approximately 0.02–0.5% incidence is reported [2]. MES can be defined by the presence of the triad of erythrocytosis, uterine fibroids, and return of RBC count to normal after surgical removal of the fibroid [3]. At present, only approximately 50 cases have been reported worldwide since the first report of MES by Thomson and Marson in 1953 [4]. Although there are hypotheses regarding the mechanism of this syndrome [5–7], its exact pathogenesis is still unclear. Here, we report the case of a huge uterine fibroid, along with findings of erythrocytosis and a history of undergoing phlebotomy procedure, as well as a detailed literature review.

## 2. Case Presentation

**Figure 1:**
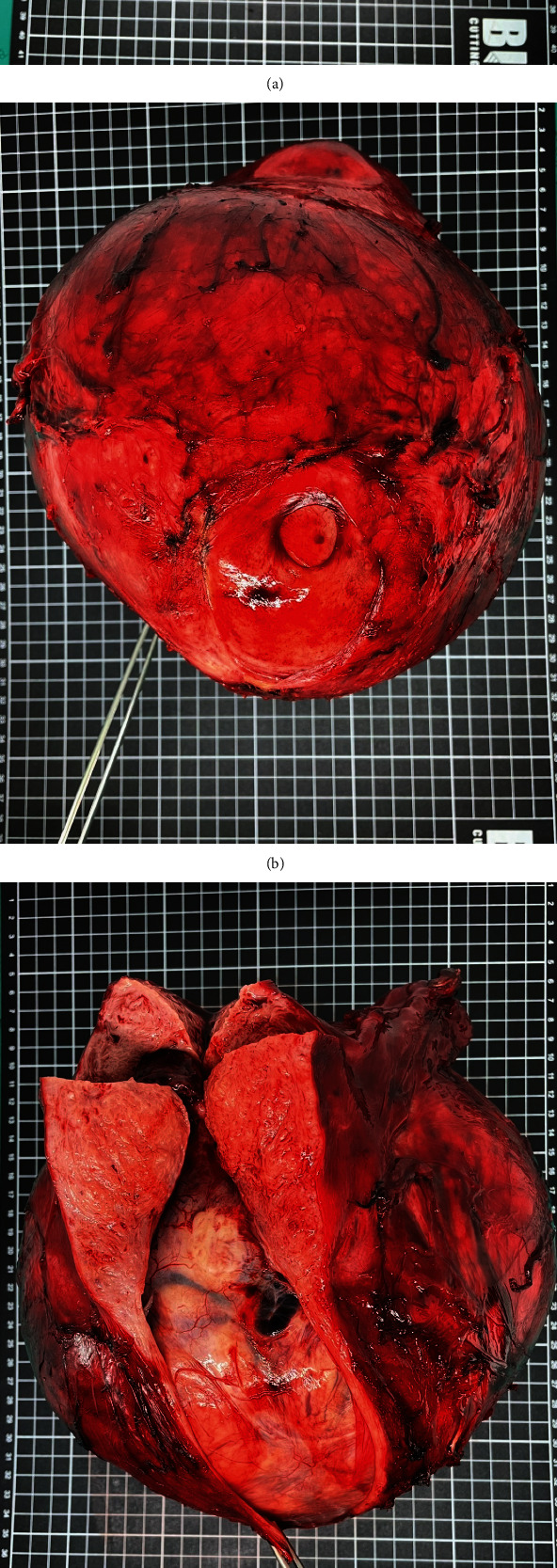
Goss specimen of a large fibroid originating from the posterior lower uterine wall (a) view from top, (b) view from below, (c) view after cut section of uterus, (d) cut surface of mass.

**Figure 2:**
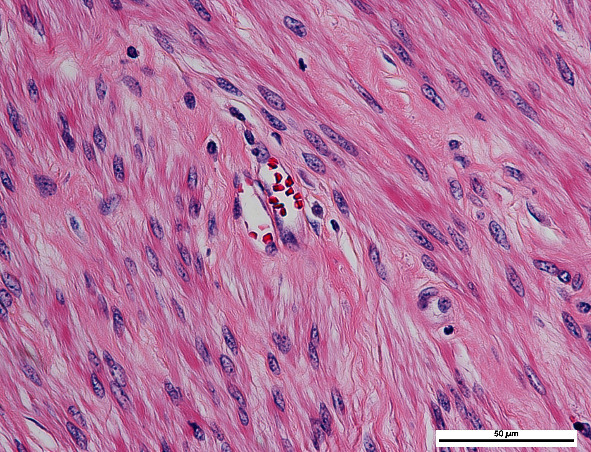
Pathology of the uterine fibroids.

A 56-year-old woman in the postmenopausal stage, gravida 2, para 2, was referred to our hospital for the evaluation and management of the uterine mass. Initially, she had a history of a palpable abdominopelvic mass for 7 years without any abnormal bleeding or compressive symptoms. She was presented to the primary hospital because of an increase in size of the mass for 1 year. Physical examination, including pelvic examination, revealed a midline pelvic mass of approximately 20 cm, and the findings of her other physical examinations were within normal limits. A computed tomography scan of the whole abdomen with subsequent magnetic resonance imaging showed a huge uterine mass of approximately 21 × 21 × 14 cm arising from the posterior wall of the uterus, as well as mild bilateral hydronephrosis due to the compression effect of the mass. Her initial laboratory investigation showed an elevated Hb level of 18.1 g/dL, a rising Hct level of 56.1%, and an RBC count of 6.52 million cells/*μ*L. The patient had normal white blood cell and platelet counts of 6,370 cells/*μ*L and 154,000 cells/*μ*L, respectively. Electrocardiogram and chest radiography findings were unremarkable. Moreover, the JAK2 V617F mutation test was negative. The underlying diseases were hypertension, diabetes mellitus, and dyslipidemia, which were well controlled with medications. Furthermore, she had a history of erythrocytosis based on incidental findings during the follow-up of her underlying disease for 1 year. She denied any symptoms of myalgia, fatigue, headache, blurred vision, or history of thromboembolism. However, she had undergone phlebotomy 1 year earlier at the primary hospital, which decreased the Hct levels from 53% to 48%. The patient's family had no history of hematological disorders. She was neither an active nor passive smoker. She did not live at a high-altitude location and did not have any lung disease. Enoxaparin 40 mg was administered to the patient subcutaneously pre-operatively, along with pneumatic calf compression to prevent venous thromboembolism. Subsequently, the patient underwent exploratory laparotomy, transabdominal hysterectomy, and bilateral salpingo-oophorectomy. The operative findings revealed a huge uterine fibroid, classified as The International Federation of Gynecology and Obstetrics (FIGO) type 6, located at the posterior lower part of the uterus, measuring 23 × 20 × 20 cm in diameter and weighing a total of 4,400 g (Figure 1). Both fallopian tubes and ovaries were unremarkable. The cut surface of the mass was grey white and well circumscribed with no area of necrosis. The pathology report consisted of a leiomyoma without evidence of malignancy (Figure 2). There were no intra-operative complications, and the intra-operative blood loss was 800 mL. On the first day of surgery, the Hb and Hct levels returned to the normal range, which were 14.1 g/dL and 43.7%, respectively. The patient was encouraged to undergo early ambulation. She was discharged from the hospital on post-operative day 3, and 40 mg enoxaparin was administered once daily for 2 weeks post-operatively. After 1 month, she was followed up at the outpatient clinic, and her hematologic parameters returned to normal values, with a Hb level of 13.5 g/dL, Hct level of 41.2%, RBC count of 4.92 million cells/*μ*L, and normal white blood cell and platelet counts. The patient appreciated the treatment outcomes.

## 3. Discussion

Several factors from outside the bone marrow can cause an increase in RBC; this condition is called secondary erythrocytosis. The known etiologies are hypoxia driven from respiratory or cardiovascular disease, renal hypoxia, exogenous erythropoietin (EPO), and pathologic EPO production from tumors, including uterine fibroids [1]. Although there has been an estimated cumulative incidence of nearly 70–80% of uterine fibroids by the age of 50 years [8], only approximately 50 cases of MES have been reported to date. According to a study by Mui et al. [5], an abdominopelvic mass was the most common presenting symptom of MES cases, followed by skin discoloration and abnormal menstrual patterns. Moreover, they reviewed 57 cases of MES and found no significant differences in parity, menopausal status, or the presence of hydronephrosis among the cases. Furthermore, the Hb level, Hct level, and RBC count were significantly reduced post-operatively, which were consistent with those of our case.

Many hypotheses have been proposed regarding the pathophysiology of MES. First, the presence of an arteriovenous shunt, intra-uterine myoma, and increasing deoxygenated blood flow may lead to an increase in the production of RBC [6]. However, this hypothesis was rejected because the arteriovenous fistula tends to affect the focal area rather than the whole hemodynamic system [3], and microscopic examination results of myoma in patients with or without MES were similar [9]. Another hypothesis is the compression theory, in which the diaphragm is compressed by a large myoma and leads to hypoxic conditions, or the renal parenchyma is affected by mass compression, which then causes an increase in EPO production [7]. Evidence supports an increase in EPO levels in MES cases [5, 9]. However, from the literature review of Mui et al. [5], the presence of hydronephrosis was not different among MES cases, as well as clinical dyspnea or hypoxia was not always seen [2, 10]. In addition, this makes this theory less reliable.

Emerging evidence shows that EPO is produced ectopically in uterine fibroid tissue in addition to the renal cortex, which can produce EPO and promote RBC production. Recently, Suresh and Rizk [9] discovered positivity of EPO immunohistochemistry in fibroid tissue, and the authors detected an increase in EPO level in blood serum, which returned to normal levels after removal of the mass. Moreover, Shu et al. [10] found that EPO immunohistochemical staining was strongly positive in the myoma tissue, which may be the cause of erythrocytosis. This led to the hypothesis that EPO production from fibroids is responsible for MES. In contrast, Asano et al. [11] evaluated 114 patients with uterine fibroid and found that EPO mRNA expression was detected in 108 patients, and the production of EPO protein was consistent with mRNA expression. However, only one patient was diagnosed with MES. They suggested that intra-tumoral EPO levels were not related to the Hb levels of patients. Moreover, there were 10 patients in whom the fibroid tissue had a higher EPO mRNA expression level than the patient with MES. Although there are many hypotheses regarding the mechanism of MES, the exact causes remain to be elucidated. Herein, despite the lack of EPO level data and immunohistochemical staining in fibroid tissue, we present a case with a complete triad of criteria for the diagnosis of MES. However, we are unable to perform EPO immunochemistry due to the unavailability in our nation. In spite of the limitation, JAK2 mutations were not detected in this patient. Therefore, polycythemia vera, a primary acquired erythrocytosis disorder [1], can be ruled out.

Erythrocytosis can lead to hyperviscosity of blood, which may increase the risk of thromboembolic events. Blood viscosity can be reduced via phlebotomy (bloodletting or venesection) [1]. In a study by Mui et al. [5], even though 51% of the patients underwent phlebotomy, 5.3% of deep vein thrombosis developed perioperatively. Unfortunately, two patients died from acute hepatic vein thrombosis and pulmonary embolism. The goal of phlebotomy is to reduce the blood volume and the risk of thromboembolic events. However, the procedure can cause adverse events such as hematoma at the puncture site, syncope, dizziness, pallor, infection, damage to surrounding tissues, and even thrombosis [12]. In our case, the patient underwent phlebotomy in the previous year without any complications. However, she did not receive definitive treatment for erythrocytosis until recently and underwent hysterectomy and bilateral salpingo-oophorectomy. In addition, despite the rarity of the disease, the diagnosis of MES should be raised in patients with unknown causes of erythrocytosis along with a pelvic mass, which can be cured by surgical removal.

## 4. Conclusion

MES is a rare but lethal gynecological disease characterized by uterine myoma and secondary erythrocytosis. The definite mechanism underlying the association between erythrocytosis and fibroids is unknown. Surgical resection is the mainstay treatment. MES should be a matter of concern in patients presenting with unknown causes of erythrocytosis and pelvic masses.

## Data Availability

The data supporting the findings of the study are included in the article.
